# Utilizing Technology to Enhance the Ecological Validity of Cognitive and Functional Assessments in Schizophrenia: An Overview of the State-of-the-Art

**DOI:** 10.1093/schizbullopen/sgae025

**Published:** 2024-11-28

**Authors:** William P Horan, Raeanne C Moore, Heather G Belanger, Philip D Harvey

**Affiliations:** Karuna Therapeutics, A Bristol Myers Squibb Company, Boston, MA, USA; University of California, Los Angeles, CA, USA; University of California, San Diego, CA, USA; Cognitive Research Corporation, St Petersburg, FL, USA; Departments of Psychiatry and Behavioral Neurosciences, and Psychology, University of South Florida, Tampa, FL, USA; University of Miami Miller School of Medicine, Miami, FL, USA

**Keywords:** technology, enhance, ecological, validity, cognitive, functional

## Abstract

Cognitive impairment is a core feature of schizophrenia and a key determinant of functional outcome. Although conventional paper-and-pencil based cognitive assessments used in schizophrenia remained relatively static during most of the 20th century, this century has witnessed the emergence of innovative digital technologies that aim to enhance the ecological validity of performance-based assessments. This narrative review provides an overview of new technologies that show promise for enhancing the ecological validity of cognitive and functional assessments. We focus on 2 approaches that are particularly relevant for schizophrenia research: (1) digital functional capacity tasks, which use simulations to measure performance of important daily life activities (e.g., virtual shopping tasks), delivered both in-person and remotely, and (2) remote device-based assessments, which include self-administered cognitive tasks (e.g., processing speed test) or functionally-focused surveys regarding momentary activities and experiences (e.g., location, social context), as well as passive sensor-based metrics (e.g., actigraphy measures of activity), during daily life. For each approach, we describe the potential for enhancing ecological validity, provide examples of select measures that have been used in schizophrenia research, summarize available data on their feasibility and validity, and consider remaining challenges. Rapidly growing evidence indicates that digital technologies have the potential to enhance the ecological validity of cognitive and functional outcome assessments, and thereby advance research into the causes of, and treatments for, functional disability in schizophrenia.

## Introduction

Since the mid-20th century, the predominant approach to cognitive assessment in schizophrenia has involved the administration of paper-and-pencil tasks by highly trained research personnel during in-person meetings conducted in a well-controlled clinical setting. This approach originated from early efforts to apply clinical neuropsychology tests, initially developed for diagnosing so-called “organic” cognitive impairment due to focal brain lesions,^1^ to people with schizophrenia. Over the past 50 years, hundreds of schizophrenia studies have utilized these types of traditional tasks or their modifications.

Chief learnings from research using traditional tasks are that cognitive impairment associated with schizophrenia (CIAS) is substantial (typically 1–1.5 SD below healthy comparison norms), spans multiple cognitive subdomains, present from before the time of psychosis onset, relatively stable across the longitudinal course of illness, detectable at attenuated levels in unaffected biological relatives, and consistently correlated with and predictive of level of functional disability.^2,3^ This corpus of research has been central to reconceptualizing of schizophrenia as a neurocognitive disorder.^4,5^ The MATRICS Consensus Cognitive Battery (MCCB),^6,7^ which is administered in-persons and is comprised of 9 paper-and-pencil tasks plus 1 computerized test that assess 7 cognitive subdomains, remains the gold standard outcome measure for clinical trials aimed at improving CIAS. Unfortunately, despite major efforts, the goal of developing efficacious treatments for CIAS remains unfulfilled.^8^

While traditional neuropsychological tasks have provided significant insights in schizophrenia and beyond, advances in neuroimaging technology and our understanding of brain-behavior relationships have challenged some foundational aspects of early neuropsychology.^9^ For example, theories of brain function localization evolved, revealing that performance on many traditional tasks often reflects multiple interacting neurocognitive circuits, with dysfunction at multiple possible locations resulting in impairments on the end-state “localized” task.^10^ This historical shift in perspective has steered clinical neuropsychological away from diagnosing focal brain pathology toward developing tools with ecologically validity-tools that measure the real-world impact of cognitive challenges on patients’ ability to function in the real-world, such as being able to work, handle finances, drive, or live independently.^11^

From a theoretical standpoint, these approaches are consistent with ecological models of cognition, which emphasize understanding cognitive processes within the context of individuals’ everyday environments.^12^ By utilizing these models, digital tools are able to capture the complexity and variability of real-world cognitive abilities, thus providing more comprehensive assessments reflective of real-life functioning.

### Goals and Structure of This Review

The goal of this narrative review is to provide an overview of digital technologies designed to improve the ecological validity of cognitive and functional assessments and their application to schizophrenia research. The review consists of 4 main sections. First, “Ecological validity: definition, methodological approaches, and relevance to schizophrenia” section provides background on ecological validity, the evolution of neuropsychological tests, and contemporary technology-enhanced assessments within this definitional framework. Next, “Digital functional capacity measures in schizophrenia” and “Portable device-based assessments in schizophrenia” sections take a deeper dive into examples of technology-enhanced ecologically focused approaches that are particularly relevant for schizophrenia research. “Digital functional capacity measures in schizophrenia” section examines digital functional capacity tasks, which use simulations to measure how well participants can perform important daily life activities, while “Portable device-based assessments in schizophrenia” section describes portable device-based assessments, including remotely administered mobile cognitive tasks, brief surveys about current activities and experiences, and passive data collection. Since the approaches covered in the second and third sections are relatively new, we aim to illustrate the range of emerging new tools in these areas and describe the current evidence supporting their utility in schizophrenia rather than providing an exhaustive review. Finally, in “Conclusions” section, we take stock of the current state of these approaches, consider ongoing challenges, and describe key future directions.

## Ecological Validity: Definition, Methodological Approaches, and Relevance to Schizophrenia

Ecological Validity is commonly defined as “the functional and predictive relationship between a patient’s performance on a set of neuropsychological tests and their behavior in a variety of real-world settings.”^13^ In this definition, the goal is indexing how testing results obtained in a separated, highly controlled artificial setting correspond to how people behave during the naturalistic course of their everyday lives in the community. For many years, critiques and debates surrounding traditional neuropsychological measures have focused on their limited ecological validity.^14–17^ Proponents of using tasks with higher ecological validity argue that there is a need to better understand how patients behave in the real-world, which is essential for creating interventions that maximize functioning more effectively in everyday life.

**Fig. 1.**
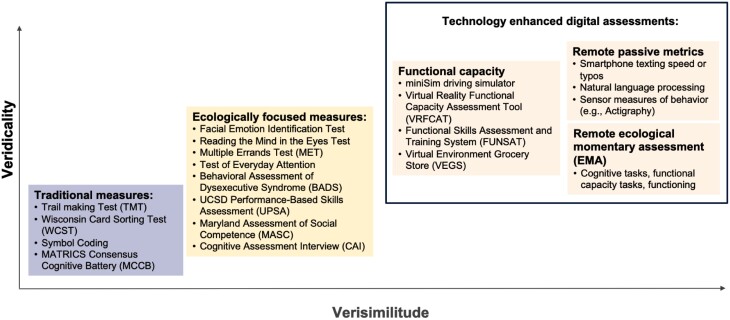
Ecological validity of cognitive assessments.

As shown in figure 1, the ecological validity of performance-based assessments is frequently evaluated in terms of 2 dimensions^13,18^:

1. Verisimilitude: “the degree to which the cognitive demands of a test theoretically resemble the cognitive demands in the everyday environment.”^13^ This overlaps with the concept of “representativeness” and is based on judgement rather than any empirical indicators. Task verisimilitude ranges from low to high levels of stimuli /activities with features of real-life.
2. Veridicality: “the degree to which existing tests are empirically related to measures of everyday functioning.”^13^ This overlaps with the concept of “generalizability” and is measured by correlation with other measures of functional outcome, which can be assessed by achievement of functional milestones (e.g., living independently, obtaining competitive employment), questionnaires, or clinical/informant ratings of real-world functioning. Task veridicality ranges from low to high levels of association with functional outcome or rehabilitation success.

The evolution of cognitive tasks since the mid-20th century can be depicted in terms of where they fall on these 2 dimensions.

### Traditional Measures

Traditional tasks from neuropsychology have relatively low verisimilitude. These tasks typically use stimuli that are simplistic, static, and neutral in emotional valence, along with well-defined instructions, to isolate cognitive constructs (e.g., executive functions, processing speed) under conditions that are as similar as possible across tests and individuals. Commonly used examples in schizophrenia include the Wisconsin Card Sorting Test,^19^ which uses colored geometric shapes to assess conceptual set-shifting, and the Digit Symbol Coding Task,^20^ which involves matching abstract symbols to numbers within a specified time period to assess psychomotor processing speed and working memory. The nature of these tasks differs from many of the complex cognitive challenges that we face in daily life, which are often poorly defined, open ended, require multi-tasking, and are imbued with social and emotional meaning as well as multiple potential distractions. Traditional tasks are amenable to standardized administration, scoring, and interpretation (e.g., with norms). Notably, many traditional paper-and-pencil administered cognitive tasks or test batteries have been adapted into computerized versions, which have several advantages in terms of computer-assisted administration to enhance standardization, automated scoring, and data management efficiencies, particularly for use in larger scale research and clinical trials.^21,22^ However, because these computerized tasks are direct digital migrations of paper and pencil tasks and are designed to be administered in a controlled (aka “ideal”) environment, their stimuli and procedures, regardless of administration format, show relatively little direct relation to many of the important cognitive demands of daily life.

Although traditional neuropsychological tests were born out of efforts to diagnose brain injuries, and ecological validity was never a consideration in their development, these tests do show some degree of veridicality. However, reviews in the general neuropsychology literature suggest a varying degree of association with everyday functioning across tasks, with many showing only a modest association to functioning.^11,17^ Their ability to predict rehabilitation success from baseline scores is generally modest, with improvements on these tasks with training/treatment typically showing limited generalizability to real-world functional gains.

These findings mirror the results in schizophrenia. In general, performance on these measures generally show modest associations (*rs* = 0.20–0.30) with real-world functioning, accounting for small to moderate variance in outcome^23^; findings are stronger for composite scores that integrate multiple subtests. Further, low generalizability of improvements in standard cognitive remediation (without concomitant psychiatric rehabilitation) to real-world functional benefits has long been an issue in schizophrenia.^24^ While functional outcome in schizophrenia is, of course, determined by multiple factors aside from cognition (e.g., motivation, psychosocial support, socioeconomic conditions, adjunctive psychiatric rehabilitation, and cultural factors to name just a few), the low verisimilitude and modest veridicality of the traditional neuropsychological tests suggest that we are not measuring all the “right stuff”^23,25,26^ to optimally understand and improve functional outcomes in schizophrenia.

### Early Ecologically Focused Measures of Cognition and Functional Capacity

Ecological tasks encompass a broad class of measures designed with the intention of more closely capturing the essence of everyday cognitive skills as they are used in real-life situations.^11,18^ These tasks have higher verisimilitude in terms of their more obviously function-focused stimuli and direct relevance to real-world activities. For example, early pencil-and-paper social cognitive measures, such as the Face Emotion Identification Test^27^ or the Reading the Mind in the Eyes test,^28^ incorporate realistic photos of social stimuli. Additional examples include the Test of Everyday Attention,^29^ which assess attentional processes using more real-world stimuli and activities (e.g., searching a map, counting elevators), and the Behavioral Assessment of the Dysexecutive Syndrome,^30^ which assesses planning and strategic thinking in relatively unstructured contexts (e.g., searching for keys, problem solving how to remove a cork from a tube while following certain rules).

In schizophrenia research, early ecological tasks have had a large impact on the field. This is particularly true for social cognition, which has become a major research topic due largely to the evidence that participants with schizophrenia show substantial impairment on pencil-and-paper or computerized social cognitive tasks (e.g., emotion perception, mentalizing) and that these tasks are more robust predictors of poor social functioning in the community than traditional nonsocial cognitive tasks.^2,31–34^ These findings have inspired novel social cognitive intervention approaches aimed at enhancing functional outcomes.^35–37^

Other early paradigms assessed performance of real-world tasks in naturalistic settings or with props or role-play scenarios to simulate real-world activities. For example, the Multiple Errands Test^38^ involves purchasing specific items, collecting and writing down specific information, and navigating to particular locations while following a set of rules in an actual shopping center. To address practical and scientific challenges (e.g., with standardized administration and scoring) of implementing these naturalistic paradigms in real-world settings, other tasks have used props or role-play tasks to assess ability to perform functional or social skills in a controlled experimental stetting.^39^ An example is the Executive Function Performance Test,^40^ which evaluates performance in the execution of 4 tasks: simple cooking, telephone use, medication management, and bill payment. Despite considerable variability across ecological measures in the extent to which their relation to real-world outcomes has been tested, reviews in the general neuropsychology literature indicate that these tests often contribute additional variance to the prediction of real-world functioning beyond traditional tests.^11^ It should be emphasized, however, that using ecological tools with higher verisimilitude does not guarantee stronger veridicality.

In schizophrenia research, an important class of early performance-based ecological tasks focuses on measuring “functional capacity.” This term refers to the ability to perform essential everyday living skills within controlled situations,^41^ including everyday living activities, work skills, and social skills. Given the close linkage of functional capacity with cognitive abilities as measured by traditional neuropsychological tasks,^41,42^ some assessment of these domains is presently required (i.e., a co-primary requirement) by regulatory authorities to demonstrate the functional relevance of interventions targeting cognition with either pharmacological or device-based computerized cognitive training (CCT) strategies.^43^ Two global assessment strategies have been proposed—structured interviews about real-world performance of cognitively demanding functional skills with patients and informants and performance on simulated assessments of functional skills.

Several interview-based measures have been developed, such as the Schizophrenia Cognition Rating Scale (SCoRS)^44^ and the Cognitive Assessment Interview (CAI),^45^ in which patients and informants are asked questions about the patient’s cognitive deficits and the degree to which these deficits impair their day-to-day functioning (e.g., cognitive challenges during everyday tasks such as conversations or using electronic devices). A major challenge with this approach is that retrospective self-reports of level of cognitive ability and functioning are notoriously inaccurate for most people with schizophrenia.^46,47^ Although high quality informant ratings can be highly accurate, many patients do not have a willing and available informant who sees them with sufficient frequency to provide meaningful ratings.^41,48^ Further, these interviews blend recollections about past performance with prospective judgments regarding abilities. As both healthy people and those with schizophrenia commonly misjudge their functional abilities in predictable (i.e., commonly overestimated) ways, this element of the assessment includes another challenge to validity.

Widely used simulation tasks in schizophrenia include the UCSD Performance-Based Skills Assessment (UPSA),^49^ which was developed in the 1990s and involves using a series of props (e.g., grocery items, a telephone, bills) to perform daily tasks such as shopping and paying bills, or the Maryland Assessment of Social Competence^50^ which involves role playing various type of interactions with a highly trained confederate (e.g., meeting a new colleague at work). While such measures avoid reliance on patient self-reports and demonstrate a closer association with cognitive test data than the interview-based measures,^48,51,52^ they have several limitations, including substantial equipment requirements, large training demands for standardized administration and scoring, a lack of alternate forms and norms, the still artificial experimental context in which they are conducted (i.e., pretend that there is an emergency and call the correct number), and content that can be outdated or even obsolete. For example, the UPSA includes tasks such as using a landline phone to dial directory assistance or paying bills with a paper check, which are no longer used in many cultures (particularly by younger people). However, a new generation of functional capacity tasks is capitalizing on technology to address the practical and scientific challenges of early tools and, hopefully, to maximize both verisimilitude and veridicality.

Advances in digital technology have had a massive impact on our lives in the 21st century. Test developers have embraced these advances and tapped into their potential to improve the ecological validity of cognitive and functional assessments, whether they are conduted within or outside of a clinical setting. Two technology-enhanced approaches with particular relevance to schizophrenia research are performance-based digital functional capacity measures, which provide increasingly realistic simulations of functional tasks for in-person assessments, and active and passive remote digital assessments, which enable cognitive and functional assessments to be conducted as people perform their regular activities in the community. The following 2 sections aim to illustrate the range of emerging new tools in these areas and describe the current evidence supporting their utility in schizophrenia.

## Digital Functional Capacity Measures in Schizophrenia

Regarding functional capacity measures, technology offers the possibility of creating ecologically valid simulation tasks with unprecedented levels of verisimilitude. These tools range from 2-dimensional, nonimmersive presentations on a computer or tablet screen, using graphics that have become increasingly sophisticated and realistic, to immersive 3-D and multisensory Virtual Reality (VR)-based tools.^53^ These computer interfaces are uniquely capable of simulating a wide range of realistic virtual environments in a safe laboratory setting, with a richness that was not previously possible.

Applications of these simulations include both assessment and intervention/rehabilitation. Additionally, these software-based programs provide a high level of experimental control, including excellent measurement precision and standardization, the ability to easily update task requirements with changes in technology, and to create alternate forms for repeated testing, and error-free scoring and data management that can meet regulatory standards. Further, VR environments have the potential to enhance task engagement through gamification, hence increasing participant motivation, thereby increasing validity and compliance. As detailed below, a range of emerging technology-enhanced simulation tasks are relevant to schizophrenia, including functional assessments such as driving, banking, using the internet, meal planning, grocery shopping. Some of these tasks are now cloud-based and fully remotely deliverable, with systematic research on the quality of their migration from in-person to remote delivery.^54^

A substantial range of measures relevant to schizophrenia are currently available, though only a subset of them have been used in this disorder. This section describes 4 measures used to varying extents in schizophrenia. The measures differ in several ways, including the type(s) of technology involved, whether they address 1 or multiple functional areas, and whether they simulate sequential daily activities (e.g., shopping) or performance of specific tasks on a more modular basis (e.g., using an automated teller machine touch screen).

### MiniSim Driving Simulator

Driving is an important functional task for people with schizophrenia given its association with autonomy and community inclusion and many people with schizophrenia do not drive.^55^ The United States Food and Drug Administration (FDA) issued guidelines regarding the use of driving simulation in clinical trials,^56^ which state that drugs with certain characteristics should be evaluated in terms of their potential effects on driving ability. The recommended means for testing driving ability are over-the-road (OTR) testing or driving simulation. Given the cost, safety issues, and other impracticalities of conducting OTR assessment, driving simulation represents a VR approach with both verisimilitude, and veridicality. The most recent reviews of the driving simulation literature^57,58^ provide evidence supporting the validity of driving simulation vis-à-vis on-road driving.

Modern driving simulators are PC-based simulators that provide a realistic automotive experience with some type of visual display of the driving environment on a screen or several screens, as well as typically a driving cockpit with some kind with a steering wheel and pedals. The “gold standard” driving simulator is a simulator that is actually an instrumented car housed within a laboratory at the National Advanced Driving Simulator (NADS) at the University of Iowa, which has established validity.^59,60^ The NADS provides a high degree of fidelity via features such as 360-degree viewing, vehicle-specific components with a 13-degree-of-freedom motion system, interactive traffic, and an actual vehicle cab. The NADS offers the highest degree of fidelity possible with a simulator, though the disadvantage is the cost and lowered efficiency associated with having an actual car cab enclosed inside a facility, as well as the engineers and other personnel that are needed to run it.

**Fig. 2.**
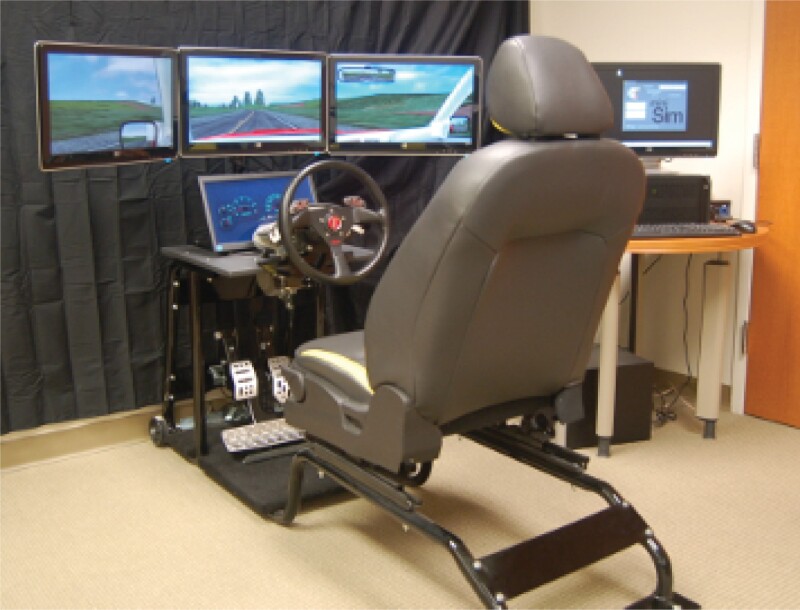
miniSim driving simulator.

A more practical driving simulator for use in clinical trials would be the MiniSim,^61^ consisting of a three-screen-wide display of the driving environment with 108-degree view of the roadway, a driving cockpit with a full-size regulation steering wheel and realistic pedals, and an actual automotive seat that meets current US National Highway Traffic Safety Administration standards (see figure 2). It has been validated against OTR driving tests^59,62^ and has been used in various clinical trials,^63–68^ though not in any published schizophrenia trials.

Only a handful of studies have used driving simulation with individuals with schizophrenia, largely with more rudimentary driving simulators than are used today.^69–71^ In these studies, individuals with schizophrenia tended to underperform relative to matched, healthy controls on most parameters, including standard deviation of lateral position (SDLP), which is the parameter frequently used in clinical trials with the most established validity.^72^ However, more modern driving simulators have revealed no^73^ or relatively small differences in those with schizophrenia, including slower speed and hinderance of surrounding drivers.^74^

Significant associations have been demonstrated between performance on cognitive tasks of concentration and vigilance and performance in a driving simulator^75^ in those with schizophrenia. Additionally, some antipsychotic (or other commonly used) medications may impair reaction time, vigilance, and driving performance in individuals with schizophrenia.^76^ While second-generation antipsychotics (SGA) typically have less adverse impact on these abilities than conventional antipsychotics,^75–77^ severity of extrapyramidal symptoms among those taking SGAs correlates negatively with driving fitness.^78^ Overall, like some of the other uses of driving simulation recommended by the FDA, this type of assessment may be best suited toward detection of worsening in performance as an adverse event of treatment in schizophrenia.

### Virtual Reality Functional Capacity Assessment Test (VRFCAT)

**Fig. 3.**
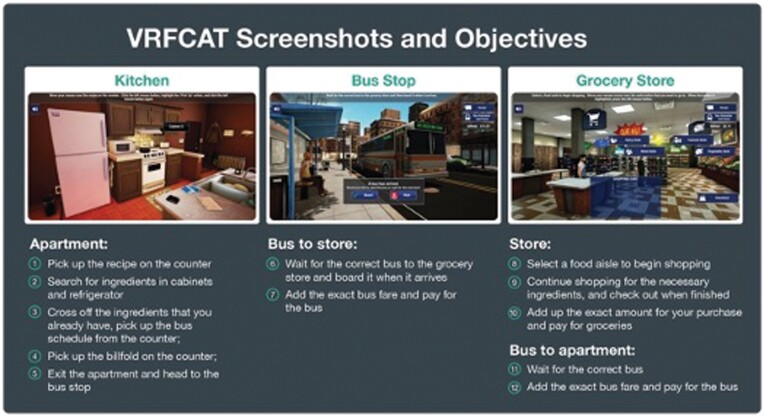
Screenshots and 12 objectives from the virtual reality functional capacity assessment tool (VRFCAT).

The VRFCAT^79^ is a semi-immersive simulation of real-world situations that is administered via a tablet interface. The VRFCAT was developed to measure an individual’s capacity to perform independent activities of daily living (IADLs) in 4 different functional abilities: food preparation, using transportation, shopping, and managing currency. The VRFCAT’s realistic, interactive and immersive environment consists of 4 mini scenarios that include navigating a kitchen, catching a bus to a grocery store, finding/purchasing food in a grocery store, and returning home on a bus. As displayed in figure 3, these scenarios were developed using immersive “first-person” gaming technology. Patients complete the scenarios through a progressive storyboard design. There are 12 different tasks or “objectives,” described in the Figure, and for each objective, the dependent variables are accuracy of performance and time to completion. For all objectives, participants who are unable to complete the objective within a pre-specified time period are “pushed” to the next objective (referred to as “Forced Progression”). The primary VRFCAT endpoint is Total Adjusted Time to complete all 12 objectives. The assessment takes approximately 30 min to complete.

Key features of the VRFCAT are that it includes 6 alternate forms to minimize practice effects, eliminates administration and scoring errors through automatic delivery of stimuli and scoring, uses automatic cloud-based data transfer, and is regulatory compliant. A large normative database is available to compute standardized scores. In addition, the VRFCAT assesses contemporary skills that are well understood and performed across many cultural contexts to facilitate translatability for multi-national trials; it has been translated and culturally adapted into more than 40 languages.

Results from the original psychometric and validation demonstrated that the VRFCAT performance in schizophrenia patients demonstrates a large separation from healthy controls (*d* = 1.20), good test-retest reliability (>0.80), good distributional properties, minimal practice effects, an interpretable unidimensional structure.^79,80^ Further, the VRFCAT demonstrated good convergent validity in terms of relations to traditional cognitive test battery performance, older measures of functional capacity (e.g., UPSA), and real-world functioning.^79^ Strong convergent validity has subsequently been demonstrated in recent-onset patients and chronic institutionalized patients.^81,82^ The VRFCAT has shown sensitivity to the effects of a computerized training interventions in schizophrenia and in older adults with Mild Cognitive Impairment.^83,84^ The VRFCAT was migrated from a device resident computerized delivery system when it was developed to tablet-based system with direct cloud capture of performance data. A comparative study of the device resident and cloud/tablet-based delivery system found very high convergence between versions.^54^ There was a slight, but systematic, performance advantage for tablet-based delivery because the tablet version does not rely on the ability to use a mouse.

Notably, a recent qualitative content validation study aimed at understanding consumer and other stakeholder perspectives on the real-world functional relevance VRFCAT^85^ found convergent evidence that patients, caretakers, and treatment providers view the content of VRFCAT as highly relevant and important for achieving and maintaining functional independence. Based on the strong psychometric/quantitative and qualitative evidence, the VRFCAT has been accepted into FDAs COA Qualification Program to be considered for qualification as a functional co-primary measure of functionally relevant changes in CIAS pharmacological clinical trials. The VRFCAT is already being used in several large international phase 2 and 3 clinical trials.

### Functional Skills Assessment and Training System (FUNSAT)

The FUNSAT^86^ involves realistic modular simulations of contemporary technology-based functional skills. FUNSAT currently has 6 technology-related functional tasks: ATM and Internet Banking, Transit Ticket Purchase, Medication Label Comprehension and Organization, Telephone voice menu on a screen-based simulation of a mobile phone, and Pharmacy Website Utilization, including Refill, On-line shopping, and home delivery (see figure 3), with more tasks currently in development and validation. Five of the 6 simulations are completely like the real-world tasks, with touch screen delivery on displays that have the same size, layout, and visual characteristics as the real-world tasks, other than the presentation of instructions for elements of task performance that would be self-generated in real-world technology utilization. The outcomes examined across the simulations have included time to completion as well as errors, in addition to examining full mastery of tasks such that inferences regarding the real-world ability to successfully execute the skills can be made. Like the VRFCAT, for all objectives, participants who are unable to complete the objective within a pre-specified time period are “pushed” to the next objective (referred to as “Forced Progression”). Three alternative forms were developed and have been shown to be similar in difficulty and psychometric characteristics and to manifest minimal retest changes.

FUNSAT, like the VRFCAT, eliminates administration and scoring errors through automatic delivery of stimuli and scoring, uses automatic cloud-based data transfer, and is regulatory compliant. Like the VRFCAT, FUNSAT assesses contemporary skills that are well understood and performed across many cultural contexts to facilitate translatability for multi-national use. In the most recent treatment study, assessments were delivered in either English or Spanish and after an in-person orientation and baseline assessment, all training and follow-up assessments were delivered fully remotely and performed at home by participants. Software features allow for rapid transition and cultural adaptation to other languages and international variation in the task stimuli and a digital migration study similar to the VRFCAT showed high convergence between the original device resident software and the cloud-based touchscreen performance across all 6 tasks.^54^

The FUNSAT has been deployed across the same populations (I.e., schizophrenia and aging-related cognitive conditions) as the prior pencil-and-paper and prop-based functional capacity measures and has been shown to manifest correlations with cognitive performance consistent with measuring related but separable performance domains.^87–89^ Healthy controls have been examined to potentially develop normative standards over time. Cross-sectional correlations with legacy paper and pencil measures of functional capacity (UPSA) as well as other digital functional capacity measures (VRFCAT) have been demonstrated as well.

**Fig. 4.**
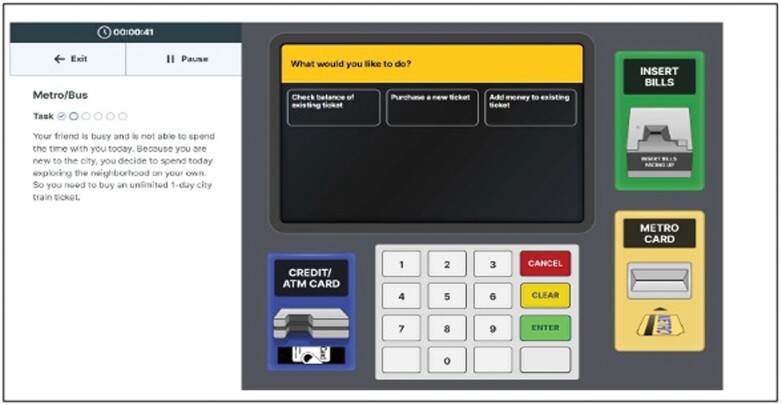
Image of the ATM task from the functional skills assessment and training system (FUNSAT).

The FUNSAT has shown sensitivity to changes associated with remotely delivered interventions, including self-administered remote cognitive and functional skills training^84,90^ (ES for FUNSAT Composite was *d* = 0.75). Post-training performance in the FUNSAT has also been shown to correlate with changes in cognition, the VRFCAT, and real-world performance of the functional skills assessed by FUNSAT (assessed on a momentary basis with EMA). The FUNSAT has not been submitted for FDA regulatory clearance as an outcome measure in pharmacological trials, but the evidence regarding its reliability, validity, and sensitivity to both impairments in illness and response to treatments directly support its applicability (figure 4).

### VR Grocery Shopping Tasks

Shopping in a grocery store represents an important functional task for people with schizophrenia. Computer-based and VR shopping measures correlate with “real life” performance in grocery stores among those with schizophrenia.^91^ Importantly, performance on VR grocery store tasks predicts variance in performance efficiency in actual grocery stores above and beyond traditional cognitive measures^91^ in those with schizophrenia. Performance on VR grocery store tasks is also related to social functioning among those with schizophrenia in ways that traditional cognitive assessment tools are not.^92^

The earliest “virtual” grocery store assessment tasks were really computer tests, rather than true VR. For example, Laroi et al.^92^ developed a computerized grocery shopping task in which participants were required to shop for a list of grocery store items using a joystick. By pressing buttons on the joystick, the participant was able to put items into a shopping cart, consult the shopping list, put items back on the shelf, move through the aisles, etc. Visual and auditory distractors were present, and various performance metrics were collected, including total time to complete the task, number of correct items, number of incorrect items, aisle redundancy, number of times the grocery list was consulted, etc. A group of 30 individuals with schizophrenia performed significantly worse than an equal number of healthy controls on most variables, with the total time to complete the task being the variable with the largest difference. A measure of social functioning (Global Assessment of Functioning) was correlated with performance on the shopping task but was not associated with performance on the traditional cognitive measures (except for a measure of cognitive inhibition), nor with the global cognitive score. This study reflected the early potential of VR-based grocery store measures for use in schizophrenia trials.

Another computer-based “virtual” grocery store task was developed by Greenberg et al.^91^ whereby participants used a joystick to navigate and manipulate their environment to find the 10 items or pieces of information (e.g., the price of an item) required. Accuracy was measured by the total number of correct items selected. Efficiency was measured by the time taken to complete the task, and the number of aisles entered above the minimum required, when using the most efficient route. In a group of 43 individuals diagnosed with schizophrenia, performance on this task was correlated with “real life” performance in grocery stores.

**Fig. 5.**
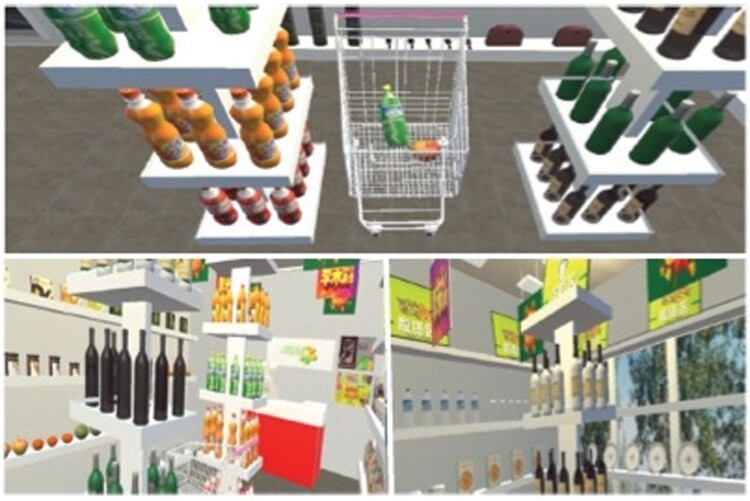
Images from the VR cognition training system (VRCTS).

Since these early studies, other more immersive VR grocery store assessment tools have been developed. Huang et al.^93^ used an immersive VR system with a helmet called the VR Cognition Training System (VRCTS, see figure 5) in 32 Han Chinese individuals with schizophrenia and 25 healthy controls. The VRCTS consists of 2 tasks which entail finding common goods on a list of items found in a grocery store and putting them into a shopping cart using a joystick. There are 4 different levels with an increasing number of items on the list. Participants must memorize the list of items, but they are able to press a button on their joystick if they forget the list and it appears again. Scores are based on the correct number of items, errors, and completion time of each task. Huang et al. reported significant differences in completion time of the 2 shopping tasks between those with schizophrenia and healthy controls, and a significant correlation between performance on the VRCTS and the composite score of the MCCB.

### Section Summary

Though a range of technology-enhanced functional cognitive measures relevant to schizophrenia have been developed,^53,94–97^ only a subset of them have been used in this disorder. The measures described above illustrate the range of real-world activities and technologies used thus far in schizophrenia. While all these measures are feasible to administer and have demonstrated separation of performance between individuals with schizophrenia and healthy controls, there are substantial differences in how extensively they have been validated and implemented in clinical trials. The more equipment and technology intensive tasks, like driving simulators and VR paradigms, have thus far been conducted in relatively small samples of people with schizophrenia by single academic research labs. The tablet-based VRFCAT and FUNSAT simulation tasks have considerably more validity evidence and documented psychometric properties, to support their use as endpoints in clinical trials. Both measures have already been successfully used in large multi-site, and even multi-country, trials in schizophrenia and other cognitively impaired populations.

## Portable Device-Based Assessments in Schizophrenia

Traditional in-person neuropsychological testing has long been the gold standard for cognitive assessment. Technological advances facilitate remote assessments of cognition and associated behaviors as participants engage in their daily activities in their natural environments. This section provides an overview of remotely delivered EMA cognitive and functional capacity measures, as well complementary sensor-based data that is passively collected.

### EMA Assessment of Cognition

Unsupervised high-frequency cognitive EMA via smartphones is increasingly used to assess cognition remotely in clinical samples, including in-persons with cognitive impairment. Utilizing an EMA approach, cognitive EMA incorporates brief cognitive assessments, either “migrated” versions of traditional tasks or purpose-built mobile tasks, into daily life. This approach allows for the collection of multiple snapshots of cognitive performance throughout the day and/or across days, enabling a deeper understanding of the ongoing patterns of daily cognition.

One of the strengths of cognitive EMA is the ability to examine both average performance and variability in performance within and between individuals across time and contexts. This method is particularly valuable as cognition can fluctuate day-to-day and is greatly influenced by context, with emerging research suggesting that intra-individual variability (IIV) holds clinical relevance and may be a particularly sensitive marker of future cognitive decline.^98–100^ Traditional neuropsychological testing makes it challenging to capture IIV over meaningful time frames and to identify other factors that may impact cognition both day-to-day and more long-term. Additionally, many traditional research methods focus on differences between individuals rather than capturing variations within individuals, an important gap that cognitive EMA can effectively address.

For example, in a sample of community-dwelling older adults, Schmitter-Edgecombe and colleagues^101^ found that IIV on a tablet-based n-back test, administered 4 times daily for a week, showed a stronger association with self-reported functional status than an individual’s average performance on the task or their global neurocognitive functioning assessed in a laboratory setting. Within-day diurnal patterns of cognitive change have also been identified. In a sample of adults with type 1 diabetes, Hernandez and colleagues^102^ administered a smartphone-based processing speed task 5 to 6 times per day for a 2-week period. They found a diurnal pattern such that processing speed was consistently slower in the morning and evening and fastest midday. This diurnal pattern of processing speed has been similarly identified in other populations, including normative data samples^103^ as well as among people with Alzheimer’s disease and related dementias. In the context of schizophrenia, where long-term trajectories of cognitive impairment vary (some people improve over time, other remain unchanged, and some worsen, e.g.,^104,105^), cognitive EMA can offer a precision health approach to individually quantifying cognition in this population.

Smartphones, with their capabilities for interactive tasks, data recording, and real-time feedback, can administer cognitive tests across a wide range of cognitive functions in an ultra-brief manner, including memory, attention, executive functions, processing speed, language, and verbal skills, visuospatial ability, motor skills, and social cognition. Furthermore, mobile assessment allows for the integration of diverse contextual factors associated with cognition, such as self-reported mood, symptoms, functional behaviors, sleep, movement patterns, physical activity, and substance use.

EMA can also be used to collect complementary information about patients’ momentary perceptions of their performance on remotely administered cognitive tasks, or even of how well they see themselves performing actual daily life tasks. For example, brief EMA surveys have been used to collect appraisals of success/failure and effort/difficulty for social and goal-directed activities.^106–108^ Unlike standard interview-based measures, which require individuals to retrospectively report their cognitive functioning over multiple weeks and often demonstrate poor convergent validity in schizophrenia,^46,47^ EMA offers a promising alternative. EMA collects self-reported data about cognition in real-time, significantly reducing the demands on memory recall. As a result, momentary judgments of performance can be captured, which can then be compared to the global judgments collected from traditional self-report measures. This comparison may help quantify how challenges in momentary judgments contribute to response biases in global self-reports.

Cognitive EMA tests can be customized to the needs to various age groups, languages, populations, and research or clinical requirements. Adherence to intensive burst assessment in observational cognitive EMA studies in diverse patient populations, including people with schizophrenia, bipolar disorder, MCI/other dementia risk, and older persons with HIV, is consistently 70% or higher (e.g.,^109–118^). These bursts have varied in frequency and intensity, with some consisting of 1 test every other day for 30 days, while others involve 3–4 tests administered 4 times daily for 7 days, with various permutations in between.

Speech samples are another example of data that can be collected remotely, and the application of computational methods such as Natural Language Processing (NLP), which can automatically conduct speech analysis and extract speech features, holds promise for the detection of specific cognitive symptoms. Language-related alterations are hallmark features in people with schizophrenia, and include reduced word and speech production, use of simpler and shorter phrases, impaired processing of complex syntax, use of more word approximations, and repetitions, and reduced semantic verbal fluency. In a recent study, Silva et al.^119^ found a decline in syntactic complexity in early psychosis, which was predictive of a diagnosis of schizophrenia. Additionally, Liebenthal et al.^120^ examined speech features and smartphone usage patterns in individuals with psychotic disorders and found that greater severity of conceptual disorganization symptoms was associated with increased speech verbosity, speech disfluency, missing smartphone data, and heightened smartphone usage during sleep time, suggesting digital measures of speech disfluency are scalable markers of clinical disorganization in this population. Integrating NLP with cognitive EMA would allow for the extraction of valuable insights from participants’ test performance as well as natural speech, as it could ultimately help track changes in language use and cognitive patterns over time in response to treatment or lifestyle changes.

Although most studies have published psychometric properties of individual cognitive EMA tests, only 2 studies have reported correlations between cognitive EMA composites with lab-based neuropsychological testing composites, resulting in correlations ranging from *r* = 0.48 to *r* = 0.53.^110,113^ Furthermore, several clinical trials are underway utilizing cognitive EMA as a more sensitive outcome measure and to reduce the Number Needed to Treat. Unfortunately, most commercially available app-based tools for cognitive assessment lack validity data for their assessments.^121^ To our knowledge, there are 11 app-based tools with published psychometric data in clinical samples^113,118,122–131^; we consider 2 examples with relatively well-developed evidence supporting their feasibility, acceptability, and validity in schizophrenia.

#### NeuroUX.

**Fig. 6.**
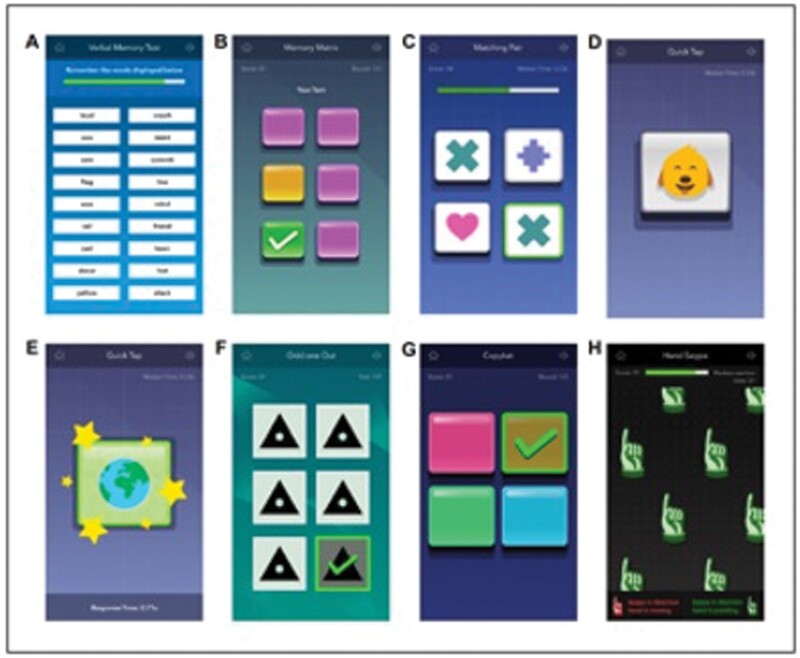
Screenshots of NeuroUX’s mobile cognitive tests: (A) memory list; (B) memory matrix; (C) matching pair; (D) quick tap 1; (E) quick tap 2; (F) odd one out; (G) CopyKat; (H) hand swype.

NeuroUX has developed a wide suite of gamified cognitive EMA tests, many of which have been extensively validated in people with schizophrenia, as well as integration of these tests with passive sensor data (see “Functional Skills Assessment and Training System (FUNSAT)” Section) and EMA surveys. Currently, NeuroUX offers 17 different cognitive EMA tests assessing cognition in the following domains: Reaction time, recall and recognition memory, visuospatial memory, working memory, processing speed, attention, executive functions, and social cognition. Normative data in a sample of 394 English-speaking, U.S. residents (aged 20–79; 50% female; 70% White; 46% iOS users; 54% Android users) is currently available for eight of these tests (figure 6).^132^ Each test takes ~1–2 min to complete. There are 2 tests from the NeuroUX battery that have the most extensive psychometric data in people with schizophrenia: the Mobile Variability Difficulty List Memory Tests (VLMT) and the EMA facial recognition test, which are described in more detail below.

The VLMT is comprised of 3 different list lengths (6-, 12-, and 18- words) and the development of this task has been previously described.^111,115^ In a study evaluating the validity of repeated administration of the VLMT among people with schizophrenia-spectrum disorders (*n* = 98) and bipolar disorder (*n* = 70), performance on the VLMT 12- and 18-word lists was positively correlated with the a laboratory assessment of the Hopkins Verbal Learning Test (*ρ* = 0.52, *P* < .001) and the UPSA (*ρ* = 0.41, *P* < .001).^111^ Adherence to the cognitive EMA protocol for the whole sample was 75.3%, and adherence did not differ by diagnostic status or correlate with cognitive or symptom variables, indicating that the task can be used by a wide constituency. No practice effects were found for the alternative forms of the 6-, 12- or 18-item VLMT lists. As expected, performance parametrically declined with longer word lists, and people with schizophrenia performed worse on the VLMT than people with bipolar disorder.

In addition to the ability to assess traditional neuropsychological domains (memory, executive functions, processing speed, etc.), NeuroUX developed a cognitive EMA test of social cognition designed for use in people with serious mental illness. In a validation study of the EMA facial recognition test, people with psychotic disorders (*n* = 86; nonaffective and affective psychosis) completed this task on their smartphones 3 times daily for a 10-day period. Convergent validity was found between performance on the mobile tasks and in-lab measures of affect recognition.^112^ Further, better performance on the cognitive EMA facial affect recognition test was associated with greater pleasure and more positive appraisals of others during social interactions.^133^ A recently published study by Parrish et al.^134^ used this task to examine relationships between suicide risk factors and facial affect recognition in a sample of 273 people with serious mental illness. This study found that real-time perceptions of threat (measured via EMA) are related to constructs relevant to suicidal ideation in this population, more-so than general facial affect recognition, which holds implications for suicide prevention work.

Bridging the gap between cognitive assessment and functional outcomes, a randomized clinical trial is currently underway (clinicaltrials.gov ID: NCT05899348) of a newly developed smartphone-based cognitive training program targeting introspective accuracy, or the second-order judgements of one’s cognitive and functional abilities as compared to objective evidence, as a mechanism by which to improve functional outcomes in people with psychotic disorders. This program, called *Improving Thinking through Everyday Self-Assessment Training* (iTEST), utilizes the VLMT and EMA facial recognition tests to conduct task-based trainings of introspective accuracy and is assesses near and far transfer of training to daily life.

#### mindLAMP.

The mindLAMP app, an open-source mental health tool, offers EMA surveys, mobile cognitive tests, and access to capturing passive smartphone data streams.^135^

Validation studies have been published with two of the mindLAMP mobile cognitive tests in people with schizophrenia: the Jewels Trail Tests A and B (smartphone versions of the Trail Making Test). These studies demonstrated the reliability and validity of these tests compared to lab-based Trail Making Tests, in the U.S. and in India, although sample sizes were relatively small (ranging from 18 to 76 people with schizophrenia).^136,137^ Another study investigated the associations between screen time and real-world cognition, as measured by the Jewels Trail Test B, among individuals with schizophrenia and found while screen time did not correlate with lab-based cognitive assessment (using the BACS), it was linked to longitudinal beta values for the Jewels B cognitive test on the mindLAMP app (*F* = 5.43, *P* < .001). Notably, this association was observed only among people with schizophrenia, as there was no significant relationships between Jewels B performance and screen time among healthy controls.^138^

Moving beyond small, single lab studies, mindLAMP’s EMA and digital phenotyping platform is currently being used as an assessment tool in the Accelerating Medicines Partnership (AMP) Schizophrenia program. This global initiative aims to develop tools that will expedite the development of effective early-stage treatments for individuals at risk for schizophrenia.

### Integrating Passive Sensor Data

Passive measures, including smartphone sensors and wearable devices, can be valuable tools in research on cognition in schizophrenia, offering unique insights into individuals’ daily lives and behaviors. Examples of data related to cognition and functioning that can be captured passively, with no effort or even awareness on the part of subjects, include movement patterns, sleep quality, social interactions, and environmental factors. Given that people with schizophrenia often exhibit deficits in their insight or impairments in recall, passive measures can provide objective and continuous assessment of their real-world functioning. Integrating passive measures with cognitive EMA can provide additional contextual information and corroborate performance data. The pairing of active and passive data can enhance the richness of data collected and well as offer a more comprehensive understanding of the dynamic interplay between symptoms, behavior, cognition, and functioning, and holds promise for informing personalized interventions.

Global Positioning System (GPS) data represents 1 passive data stream that has demonstrated validity as a metric of functioning in people with schizophrenia. For instance, the severity of avolition can be characterized by combining EMA surveys and GPS coordinates to determine if an individual spends most of their time home, alone, and engaged in unproductive activities such as pacing, smoking, watching TV, resting, sitting alone, or doing nothing. Numerous investigations have found strong convergence between self-reported (EMA) and GPS-based location measures in individuals diagnosed with schizophrenia, with both data streams significantly correlating with clinician ratings of negative symptoms (e.g.,^139,140^; see Daniel et al., 2023 for a comprehensive review of remote assessment of negative symptoms of schizophrenia^141^).

Another source of passively collected data is meta-data is derived from interactions with mobile devices. This can include number of text or e-mail messages sent, information searches, and typing speed. These indicators are also easily obtained and can be used to predict other elements of momentary behaviors, including sustaining stable daily routines^142^ or larger issues such as impending relapse.^143^ The interaction between different elements of passively measured information streams can also be monitored remotely and provides the possibility of customized interventions based on continuous assessment of passively collected data.

Overall, the integration of passive measures with cognitive EMA holds significant promise in advancing our understanding of cognitive processes in schizophrenia and could provide valuable insights into real-world manifestations of symptoms and functioning in people with schizophrenia. Both NeuroUX and AMP-SZ include access to collection of passive data streams in their platforms, to be utilized in conjunction with the mobile cognitive tasks or independently, thus streamlining the data collection process. To date, few studies have used digital phenotyping approaches in people with schizophrenia (see Lane et al. for a review^144,145^), but there is promise that this method could enable a more holistic approach to assessing cognitive functioning, as well as enhance our ability the link cognitive abilities and daily functioning.

### Other Remote Assessment Applications in Schizophrenia

The lessons learned in the COVID pandemic have found that some interview and performance-based functional capacity measures can be assessed on a remote basis. For informant based functional capacity interviews, such as the SCoRS, the CAI, or interactive verbal-only strategies (such as the SSPA), video-conferencing strategies have been shown to be easily adaptable. Both for participant and informant data collection, there is no requirement for manipulation of props or stimulus items, as are required for paper and pencil neuropsychological tests or functional capacity assessments. In fact, recent published studies have used data collected in-person or with video conferencing for both the CAI and SSPA, with no differences in performance associated with data collection modality.^146^

Regarding performance-based VRFCAT and FUNSAT functional capacity used in schizophrenia and other cognitively impaired populations, the VRFCAT has been delivered exclusively on an in-person basis. Even in studies with remote training, the VRFCAT assessment was performed in-person.^83^ In contrast, the current version of FUNSAT is cloud-based and fully remotely deliverable. In a recent validation study of the FUNSAT software in MCI and cognitively unimpaired individuals, remotely captured performance on the FUNSAT was found to be feasible, with over 90% adherence and valid data collected in MCI and cognitively unimpaired population.^84^ Further, both remotely delivered FUNSAT assessments and in-person VRFCAT assessments were sensitive to the effects of a remotely-delivered combined CCT and skills training intervention and were found to improve by very similar amounts from baseline to the end of training (ES for FUNSAT Composite was *d* = 0.75; ES for VRFCAT total adjusted time to completion: *d* = 0.64).^84,90,147^ The FUNSAT system with remote delivery has been used as an outcome measure primarily in trials of MCI. Large-scale clinical initiatives in SMI populations are underway and will provide similar feasibility data for remote assessments in this population.

### Section Summary

Collectively, these findings demonstrate remotely deliverable cognitive EMA and functional capacity measures, and even fully remote cognitive and skills training interventions, have proven feasible and acceptable in participants with schizophrenia and in other cognitively impaired populations. Use of cognitive EMA may allow for a deeper understanding of daily cognitive functioning as well as intra-individual variability over time. While most commercially available app-based tools for cognitive assessment lack validity data, 2 exceptions are the NeuroUX battery and mindLAMP, which have both produced initial validity data in individuals with schizophrenia. Remote collection of complementary passive data, such as GPS, and voice, also demonstrates initial validity in those with schizophrenia. In addition, functional capacity interviews are amenable to administration outside of traditional research settings via video-conferencing technology in schizophrenia, and initial studies support the validity of remote, unsupervised administration of the performance-based FUNSAT in people with MCI.

## Conclusions

Cognitive and functional capacity assessment developers have embraced technological advances in the early 21st century. This has led to a surge of innovative measures and methods that show considerable promise for enhancing the ecological validity, including both verisimilitude and veridicality, of assessments conducted both inside and outside of a clinical research setting. There is solid evidence for the feasibility and initial validity of all the types of measures considered in schizophrenia. Furthermore, development and validation of the VRFCAT, FUNSAT, and some remote cognitive EMA measures are already at a relatively advanced stage. Driving simulation has also been used extensively in clinical trials, though not specifically in schizophrenia. The hope is that more ecologically valid cognitive measures can be used in observational studies to help us identify more functionally impactful targets for psychosocial and pharmacological interventions in schizophrenia. Ideally, these tasks could also serve as sensitive endpoints in clinical trials to evaluate the efficacy of novel interventions aimed at improving functional outcomes.

### Remaining Challenges

The proliferation of technology-enhanced measures reflects an exciting new chapter in the history of cognitive and functional outcome assessments in schizophrenia. Whether these measure will ultimately address key limitations of traditional neuropsychological tasks remains to be seen. In the context of rapidly emerging and alluringly marketed new technologies, validation needs to catch up with development. For the promise of new ecological measures to be fully realized, the field will need to address practical, scientific, and regulatory challenges.

Practical challenges include factors such as accessibility, compatibility, costs, staff training needs, and participant support needs for new technology-enhanced assessments. For example, although VR paradigms offer the potential for in-clinic assessments with unprecedented verisimilitude, their development and uptake have been hampered by expensive specialized equipment and resource requirements. Major advances in terms of verisimilitude, compactness, hardware-software integration, and reduced costs have increased the practicality of conducting larger scale VR validation studies in recent years.^148^ However, further method development is clearly needed for scalable implementation of VR assessments in large multi-site trials.

For unsupervised cognitive EMA and passive remote measures, additional practical challenges include attributability, dealing with nonadherence and missing data, inter-operability between different hardware and operating systems, and ensuring data security and privacy.^149^ Notably, due to privacy concerns, some manufacturers and regional legislative bodies are making it more difficult to collect certain types of data (e.g., GPS) from smartphones. Further, although remote digital measures have the potential to substantially expand geographic and participant diversity representation in clinical trials, it is critical to consider potential access and acceptance barriers associated with factors such as age, socioeconomic status, and cultural characteristics.

From a scientific perspective, it is critical for researchers to be clear about how they operationalize “ecological validity” when developing technology-enhanced methods. Recent reviews document the diverse types of evidence that have been used to support claims of a test’s ecological validity (e.g., predict daily functioning, differentiated clinical groups, correlated with other cognitive tests, has face validity).^150–152^ Consequently, the term has been criticized for being conceptually vague and potentially misleading, emphasizing the need for researchers to specify the functional context of the cognitive processes in which they are interested.^150^ In schizophrenia, the goal of using ecologically valid tests is typically to more precisely identify the determinants of daily life functional difficulties (i.e., veridicality together with verisimilitude) so we can develop and test interventions that more effectively target them. For technology-enhanced functional capacity measures used in clinical research settings, the scientific requirements are largely the same as for any new performance-based outcome assessment: strong quantitative evidence for their psychometric characteristics and convergent/discriminant validity, as well as qualitative evidence supporting their content validity, is required for acceptance by regulators.^153,154^ Along these lines, test developers and regulators will need to agree on what constitutes a “ground truth” daily life functioning metric to establish their validity for use in clinical trials.

However, efforts to validate new technology-enhanced methods raise some unique complications, particularly for remotely collected assessments. For unsupervised cognitive EMA and passive remote measures, all the standard psychometric and validation requirements for in-clinic assessments are applicable for regulatory acceptance.^149^ Although legacy cognitive and clinical measures created decades ago are often considered gold standards for validation from a regulatory perspective, it is possible that novel remote measures can provide superior precision or even capture qualitatively distinct aspects of functioning, resulting in relatively lower convergence with traditional measures as compared to the typical standards applied to evaluating convergent validity among traditional clinic-based measures. What is an appropriate level of convergence with traditional gold standard clinical measures? In addition, passive sensor-based measures collect very large quantities of data that are typically processed using (often proprietary) artificial intelligence-based algorithms to create clinically meaningful outcome variables. These AI-derived metrics will need to be sufficiently transparent and understandable for regulatory acceptance.^155,156^

### Looking Forward

Looking forward, we expect the current proliferation of technology-enhanced ecologically valid assessments is unlikely to slow down. We anticipate expansion of clinic-based VR approaches to other functionally important areas, such as using chatbots and avatars, which are increasingly sophisticated, to provide veridical assessments of social cognitive processes. The trend toward increasingly hybrid in-clinic/remote assessment approaches is likely to continue; in fact, fully remote trials that provide both computerized cognitive remediation or functional skills training and clinical assessments almost entirely outside of a conventional clinical research setting are already happening.^84,90,147^ Finally, despite the challenges associated with remote passive metrics, we expect increased development efforts in this realm due to the appeal of identifying objective indices of real-world cognition and functioning that involve no deliberate effort aside from wearing or carrying a digital device.
